# The effects of a mobile application for patient participation to improve patient safety

**DOI:** 10.1111/hex.13503

**Published:** 2022-05-11

**Authors:** Nam‐Ju Lee, Shinae Ahn, Miseon Lee

**Affiliations:** ^1^ College of Nursing Seoul National University Seoul South Korea; ^2^ The Research Institute of Nursing Science Seoul National University Seoul South Korea; ^3^ Department of Nursing Wonkwang University Jeonbuk South Korea

**Keywords:** mobile applications, patient education, patient participation, patient safety

## Abstract

**Background:**

Patient participation in patient safety activities in care processes is a fundamental element of safer care. Patients play an important role in preventing patient safety incidents and improving health outcomes. Therefore, healthcare providers need to develop and provide educational materials and actionable tools for patient participation.

**Objectives:**

This study aimed to develop a mobile application for health consumers' participation and evaluate the effect of the mobile application on improving health consumers' participation in patient safety.

**Methods:**

A quasi‐experimental design was adopted. We developed a mobile application on the basis of a needs assessment, literature review, compilation of patient safety topics, and validity testing of the application. The target population included Korean adults aged between 30 and 65 years who had visited a medical institution more than once within the most recent 6 months. The intervention group received patient participation training by using the mobile application, Application for Patient Participation in Safety Enhancement, for 2 months. The primary outcome variables were patient safety knowledge, self‐efficacy of participation, willingness to participate and experience of patient participation in patient safety activities. End‐user satisfaction was assessed using a questionnaire. To assess participants' experiences with the intervention, qualitative data were collected through a focus group interview and open‐ended responses to an end‐user satisfaction survey.

**Results:**

The intervention group (*n* = 60) had significantly higher overall average scores than the control group (*n* = 37) with regard to patient safety knowledge (*p* < .001), self‐efficacy of participation (*p* = .001), willingness to participate (*p* = .010) and experience of participation (*p* = .038) in the post‐survey. The total mean end‐user satisfaction score was 3.56 ± 0.60. The participants expressed the realization that patients could play an important role in improving patient safety.

**Conclusions:**

This study demonstrated that educating health consumers through a mobile application with useful information improves patient participation in patient safety activities. Educational materials and patient participation tools could motivate health consumers' health‐related behaviours.

**Patient or Public Contribution:**

Patients were involved during the programme development and evaluation.

## 1 INTRODUCTION

In recent years, patient participation has emerged as a major factor in improving patient safety and health outcomes. There are various definitions of patient participation, and the term is also used synonymously and interchangeably with patient engagement and patient involvement in the literature.^1^, ^2^, ^3^ Patient participation is characterized by information flow between patients and providers, as well as an active role of patients in care decisions.^4^ Patient participation from patients' perspectives relates to receiving explanations and having knowledge of (a) plans, (b) where to turn for help and (c) what to do to feel well.^5^ Previous studies have identified patient participation in healthcare as occurring at three levels: micro (direct care), meso (organizations) and macro (society and government).^1^, ^2^ Each level is characterized by different types and activities of patient participation and can range from individual participation in the primary care process to participation in policy making.^1^, ^2^, ^3^ The National Health Service (NHS) has developed a framework for patient participation consisting of three levels of participation (information, involvement, and partnership or shared leadership) and levels of the healthcare system (one's own care, service providers and the system).^4^ At higher levels, patients participate more actively in the care process and have greater shared power.^4^, ^6^ In that aspect, patient participation can be seen as a strategy to achieve patient‐centred care,^2^ which can affect patient safety.

As part of improving patient safety, which prevents avoidable harm in healthcare, patients must be informed and knowledgeable healthcare partners who are empowered to have a voice and share their decisions.^3^, ^7^, ^8^ Therefore, the role of patients and their caregivers in active participation in their healthcare has been emphasized as a prerequisite for patient safety. In most stages of patient care, patients are likely to contribute to their own care to prevent adverse events.^9^ Previous research has shown that patient participation in healthcare has positive impacts on healthcare quality and patient safety.^3^, ^10^, ^11^, ^12^ For example, patients can report an error when they notice it, and they can ask healthcare providers to wash their hands to prevent infection.^10^ Thus, patients can play an important role in reducing patient safety incidents, as well as having a direct impact on better health outcomes.

In the United Kingdom, a report on patient safety pointed out that patients' codesign, coproduction and participation in initiatives are important for preventing patient safety incidents.^13^ Though recognition and encouragement of the patient's active participation in their care are growing, it can be difficult and unfamiliar for patients to actually participate.^14^ Previous research as part of our project examining the factors influencing patient participation in patient safety activities showed that health consumers' recognition of the importance of patient participation was high, but their experience of participation in patient safety activities was limited.^15^ Health consumers reported that they wanted education programmes reflecting their diverse needs with regard to participation in their care process.^15^ Interventions, including safety‐related information and education, are considered important components of promoting patient participation.^10^, ^16^ However, there is a lack of educational programmes that provide information on what patients should do or how they can participate in safety activities. Therefore, it is necessary to provide relevant information and education to health consumers through interventions that are based on their educational needs.

Several collections of educational materials have been provided by international patient safety organizations including the Agency for Healthcare Research and Quality's (AHRQ's) 20 Tips to Help Prevent Medical Errors^17^ and Australian Commission on Safety and Quality in Health Care's (ACSQHC's) Top Tips for Safe Health Care.^18^ While educational materials for health consumers do exist, most of these materials address specific aspects of patient safety. A few studies have developed patient participation programmes such as education programmes for medication safety, hospital‐acquired infection prevention, surgical checklists or hand hygiene.^11^, ^19^

To broaden the range of patients who can participate in their care process to prevent errors and lead to better health outcomes, it is necessary to expand the research population to include health consumers who visit the hospital. Some studies have conducted interventions for patient participation and evaluated their effectiveness.^10^, ^20^, ^21^ However, these studies were more focused on hospitalized patients^20^ or patients with a certain disease.^21^

Interventions using information technology, such as mobile applications, have been shown to be effective in terms of increasing patients' self‐management, facilitating communication between patients and healthcare providers, and enhancing patients' knowledge, while overcoming the temporal or spatial constraints of other interventions.^20^, ^21^, ^22^ Nearly 91.0% of South Koreans own a smartphone, and 93.7% use the internet for knowledge acquisition or searching for information, including online health information, via mobile devices or computers.^23^ Therefore, using a mobile application should be an effective way of improving health consumers' knowledge and perceptions of patient safety activities, and their associated behaviours. Although some mobile applications have been developed for patient safety education,^20^, ^24^ these applications have mostly focused on the prevention of surgical errors and have not addressed a wide range of patient safety activities for patient participation.

The aim of this interventional study was to develop a mobile application for health consumers' participation in patient safety activities and evaluate the effect of the mobile application on improving health consumers' patient participation. To evaluate patient participation, this study assessed health consumers' patient safety knowledge, self‐efficacy of participation, willingness to participate and experience of patient participation in patient safety activities. This study also investigated participants' satisfaction and experiences with the application.

## 2 METHODS

### 2.1 Study design

This study consisted of two stages of system development through a sequential development process and evaluation of the effectiveness of the system. This study used a quasi‐experimental design to evaluate the effectiveness of the Application for Patient Participation in Safety Enhancement (APPSE).

Patients participated in the following development and evaluation phases: (1) evidence collection: A systematic review of the evidence on interventions for patients' and families' participation in patient safety; (2) needs assessment: A health consumer survey assessing their perception and experiences of patient safety and focus group interviews exploring health consumers' experiences of patient participation in patient safety and their need for education on patient participation; and (3) an evaluation study: A quasi‐experimental study to evaluate the developed APPSE and a focus group interview to explore users' experiences with the APPSE. The results of (1) and (2) have been described in detail elsewhere.^8^, ^15^

### 2.2 Participants

The target population comprised Korean adults who had visited a medical institution within the most recent 6 months. The inclusion criteria were as follows: (1) adults aged between 30 and 65 years who had visited a medical institution more than once within the most recent 6 months; (2) who agreed to participate in the study and provided signed online informed consent; and (3) who owned a mobile phone capable of receiving text messages and who utilized the application using computer/mobile devices. Adults who were employed at a medical institution and provided health services as healthcare providers were excluded, as it was felt they may represent a biased view of participation. The sample size was calculated using G*Power 3.1,^25^ and it was determined that 84 participants (experimental group = 56, control group = 28) would be required to detect differences in the post‐survey outcomes of patient participation (patient safety knowledge, self‐efficacy of participation, willingness to participate and experience of patient participation) of the two groups, with a 2:1 ratio with a two‐sided type I error of 0.05, an effect size of 0.68 and power of 80%. The effect size was based on a previous study^24^ that evaluated the effectiveness of an application for surgical patients' patient safety. Since a higher dropout rate was expected in the experimental group receiving the intervention than in the control group without the intervention, a 2:1 ratio assignment was used in this study. With reference to the results of previous studies,^26^, ^27^ we expected a dropout rate of 30% and aimed to recruit 110 participants.

### 2.3 Development of the APPSE

**Figure 1:**
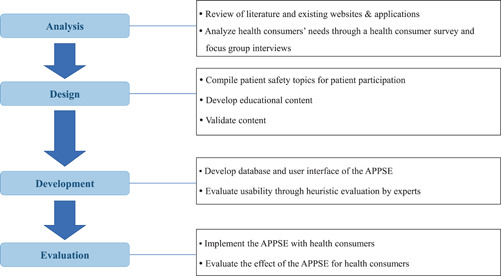
Stages of programme development. APPSE, Application for Patient Participation in Safety Enhancement

The APPSE for health consumers' participation in patient safety was developed according to the sequential steps of analysis, design, development and evaluation (Figure 1).

#### 2.3.1 Analysis phase (needs assessment and literature review)

To develop the APPSE, a systemic review of the literature on patient and family participation in patient safety activities was performed.^8^ We also explored websites of the international organizations and existing applications that provide information or materials on patient safety. We examined health consumers' perceptions and experiences of participation in patient safety activities, as well as their needs related to patient participation, through a health consumer survey and focus group interviews in previous research^15^ by our project team. From that study, we found that health consumers wanted to be educated on various patient participation topics. We categorized them as follows: ‘patient rights’, ‘disease and diagnosis’, ‘treatment’, ‘surgery and medical testing’, ‘medication’, ‘patient advocacy’, ‘patient question checklists’, ‘manuals for empowered patients’ and ‘error reporting’.

#### 2.3.2 Design phase (content development for APPSE)

Based on the results of the health consumers' requirements analysis, a literature review, and an examination of existing patient safety websites and applications, the research team developed the APPSE framework to compile the educational content, which consists of 12 patient safety topics with four safety competencies (SAFE: Speaking up, Asking questions, Finding health information and Engaging in the healthcare process) in patient safety activities that patients could participate in during their care process. The four SAFE competencies were classified based on the level of participation of the NHS framework. For content development, the research team used a total of 63 collections of educational materials addressing patient participation in patient safety provided from 16 international organizations, among them The Joint Commission,^28^ the World Health Organization,^29^ the AHRQ^30^ and the ACSQHC,^31^ after receiving permission for use from each organization. Through regular meetings, the research team reviewed the educational materials, translated the contents into Korean and modified them using terms easily understandable to health consumers. We sorted the educational materials according to the APPSE framework. The final educational content was developed after a validity review by an expert panel comprised of one doctor, two nurses and one pharmacist.

#### 2.3.3 Development phase (development of the application)

After the framework of the APPSE was designed based on the outcomes of the analysis, the research team developed user scenarios and user diagrams of how the users utilized the APPSE functions and performed the tasks through brainstorming in iterative project meetings. The system database and user interface were designed according to the use case diagrams. The server configuration used XpressEngine^32^ and a MySQL database. The APPSE was designed with a responsive web design for use on both the web and mobile devices. The research team provided a layout for the interface design, and an app programmer developed the programme following the design.

**Figure 2:**
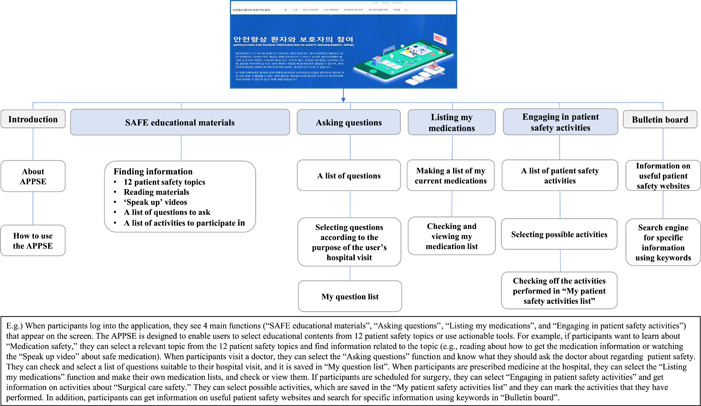
General overview of ‘Application for Patient Participation in Safety Enhancement’ (APPSE)

The APPSE consists of the following four main components (Figure 2): (1) The ‘SAFE educational materials’ page provides SAFE educational materials for 12 patient safety topics according to four patient safety competencies. The 12 patient safety topics that patients and families can encounter in healthcare institutions were patients' rights, patient–provider communication, patient advocate, error prevention, patient identity, medical tests, medication safety, surgical care safety, infection prevention, prevention of falls/bed sores/blood clots, preparation for hospitalization and transition of care. On each page of the SAFE educational materials, the user can find educational information related to the chosen topic, including reading materials, the list of ‘speak up’ videos to watch, and the list of questions to ask or activities to participate in. (2) ‘Asking questions’ is composed of listing my patient safety questions and viewing them. The system provides a list of patient safety questions and a user can select questions guided by the system according to the purpose of the user's hospital visit. This function helps patients to prepare questions in advance of visiting a hospital. (3) ‘Listing my medications’ contains the functions of making a list of my current medication and checking and viewing my medication lists. This function helps patients to update their current medication list and use it when consulting doctors and pharmacists. (4) ‘Engaging in patient safety activities’ is designed to provide a list of patient safety activities that the patient can participate in, depending on the patient's situation. A user can select topics that they can participate in and mark the activities that they have performed. There is a ‘Bulletin board’ that provides useful patient safety websites. Table 1 presents the structure of the SAFE educational content for 12 patient safety topics and the components of the APPSE.

**Table 1:** Structure of the SAFE educational content for 12 patient safety topics in the APPSE

Patient safety topics	Description		Educational contents and components according to SAFE competency				Added based on	
		*Competency*	Speaking	Asking	Finding	Engaging	Patients' needs assessment	Literature and expert review
		*Educational contents:*	List of speak up videos	List of questions to ask	Reading materials	List of activities to engage in		
		*Component in the APPSE:*	SAFE educational materials	Asking questions/SAFE educational materials	SAFE educational materials	Engaging in the healthcare process/SAFE educational materials		
Patients' rights	All patients have the right to receive appropriate health and medical treatment services to protect and improve their own health. This includes the right to health, the right to know about health and medical services, the right to decide on health and medical treatment services and a guarantee of confidentiality.		•	•	•		•	•
Patient–provider communication	Patient–provider communication is communication exchanged between patients and providers in healthcare. Preparing to communicate with providers can help you communicate more effectively, which allows you get safer care and make better decisions when making treatment plans.		•	•	•	•		•
Patient advocate	Patient advocates are people who help and support patients in the healthcare system. They can help you communicate with providers and make medical appointments. They also can be with you for your care, treatment and examination, ask questions and participate to make better decisions.		•		•		•	•
Error prevention	Medical errors can occur any time, anywhere in the healthcare system. To keep yourself safe, you should actively participate as a member of your healthcare team. You can gain better outcomes and prevent patient safety events by participating more in decision‐making and healthcare.		•	•	•	•	•	•
Patient identity	Patient identification helps to provide safe and accurate treatment and healthcare services to patients by accurately identifying patients in all treatment processes.				•	•		•
Medical tests	Medical tests help to determine a patient's condition, make a diagnosis, plan treatment and monitor whether treatment is effective. (But sometimes, the wrong tests are ordered, and doctors get results too late, delaying the best treatment.)			•	•	•	•	•
Medication safety	Medication errors can occur anywhere in the process from medication prescription to medication use, which can lead to minor to life‐threatening side effects. Medication safety means being free from accidental events mentioned above. Knowing accurate information about your drug helps prevent errors.		•	•	•	•	•	•
Surgical care safety	Errors related to surgery can occur anywhere before, during and after surgery, and the types of patient safety events that can occur are also very diverse. It is important to actively participate to prevent patient safety events that may occur in surgery and to know accurate information about surgery.		•	•	•	•		•
Infection prevention	Healthcare‐associated infection refers to infections caused by the spread of bacteria from the patient himself or herself or other patients while receiving appropriate healthcare at the hospital, and are preventable patient safety events. In particular, patients with compromised immune system are easily exposed to infection, and infection can be fatal; therefore, attention is needed.		•	•	•	•		•
Prevention of falls/bed sores/blood clots	Falls are a common patient safety event and can occur anywhere including hospitals and patients' homes. Patients are physically vulnerable and may become tired or sleepy from drugs or other influences. Patients and families can improve patient safety by participating in fall prevention.		•		•	•		•
Preparation for hospitalization	Hospitals are a very complex environment and one can meet many people there, so once hospitalization is decided, preparations for hospitalization are required. Well‐prepared hospitalization can help you recover quickly during hospitalization and allow you to receive safer care.			•	•	•		•
Transition of care	When you are discharged from the hospital, you need to be cared for until full recovery at home or in a nursing home. To take care of yourself, you need to prepare many things for discharge. If you know and prepare what you need to know after discharge before you leave the hospital, you can improve the safety of your care.		•	•	•	•		•

Abbreviations: APPSE, Application for Patient Participation in Safety Enhancement; SAFE, Speaking up, Asking questions, Finding health information and Engaging in the healthcare process.

To evaluate usability, we conducted a heuristic evaluation, which is the most widely adopted and efficient expert‐based usability evaluation method.^33^ Nielsen's heuristics are broad rules of thumb for designing new rules and guidelines in user interfaces; these heuristics are still valid and are widely used today.^34^ Three to five experts are sufficient to achieve optimal effectiveness in identifying usability problems.^35^, ^36^, ^37^

Three experts who specialized in nursing informatics and had experience in developing applications were involved in the heuristic evaluation, which used a tool modified for the evaluation of the APPSE app based on Lee's^38^ heuristic evaluation checklist. The heuristic evaluation tool included written instructions with information about evaluation, the list of tasks to carry out using the APPSE app and a heuristic checklist based on Nielsen's 10 heuristic principles (consistency, metaphor, visibility, efficiency, memory, freedom, minimalism, aesthetics, error prevention, helpfulness) with a 5‐point scale (0 = ‘I don't agree that this is a usability problem at all’, 1 = ‘Cosmetic problem only: does not need to be fixed unless extra time is available on project’, 2 = ‘Minor usability problem: fixing this should be given low priority’, 3 = ‘Major usability problem: important to fix, so should be given high priority’, 4 = ‘Usability catastrophe: imperative to fix this before product can be released’). Mobile and webpage links were provided to each expert and they independently rated the APPSE app. The mean score of each heuristic was 0.38 (SD = 0.25). Based on the heuristic evaluation, the APPSE app was revised to increase letter size and spacing in consideration of readability, and the final version was developed.

### 2.4 Intervention (evaluation of the APPSE)

Participants in the intervention group received patient participation training by using the APPSE through the webpage or mobile application for 2 months. The research team sent text messages to participants before starting the introduction that contained an instruction brochure on how to use the APPSE. They were guided to log into the application and use the APPSE. After registering for the system, participants were free to log in on both web and mobile and use the APPSE at any time during a 2‐month period. To encourage use of the APPSE, a brief summary of an educational topic in the APPSE and related links were delivered to participants by a text message once a week. Also, user log analysis was performed, and a text message was sent once a week to encourage use by participants with a low usage rate. The participants in the control group were not provided with an intervention during the study process to avoid bias.

### 2.5 Measure

The primary outcome variables measured to evaluate the effect of the intervention were patient safety knowledge, self‐efficacy of participation, willingness to participate and experience of patient participation in patient safety activities. The secondary outcomes were users' satisfaction and the user experience with the application.

#### 2.5.1 Baseline characteristics

The baseline characteristics included the health consumer's demographics (age, gender, education, marital status, employment and monthly income), health status (the number of chronic diseases) and use of medical institutions (the number of and reason for visits to a medical institution, experience of hospital admission, type of medical institution frequently visited and existence of an accompanying caregiver), experience of a medical error, experience of patient safety education and experience of patient participation education.

#### 2.5.2 Primary outcome measures

##### Patient safety knowledge

Patient safety knowledge was measured using a questionnaire that consisted of 10 items about the perception of patient safety with a 5‐point Likert scale (1 = disagree strongly, to 5 = agree strongly) developed by An et al.^16^ Permission to use the questionnaire was obtained from the authors. We then added two items (‘I know my rights as a patient for treatment and health care’ and ‘I know the necessity and role of a patient advocate’) from the relevant literature.^3^, ^39^ Thus, the final questionnaire comprised 12 items. The Cronbach's *α* value of the original questionnaire was .922,^16^ and that of the finalized questionnaire in this study was .934.

##### Self‐efficacy of participation, willingness to participate and experience of patient participation

Patient participation was measured using a 13‐item scale developed by Lee et al.^15^ The survey comprised 13 items in total, which assessed specific safety‐related activities in which patients can participate while visiting a medical institution. We measured three areas (self‐efficacy of participation, willingness to participate and experience of patient participation). Four‐point Likert scales were used to assess self‐efficacy of participation (1 = not at all, 2 = sometimes, 3 = often, 4 = always), willingness to participate (1 = not at all, 2 = somewhat likely, 3 = likely, 4 = very likely) and experience participating in patient safety activities in a medical institution (1 = not at all, 2 = sometimes, 3 = often, 4 = always). The Cronbach's *α* values of the three sections in this study were .916, .879 and .946.

#### 2.5.3 Secondary outcome measures

##### End‐user satisfaction

End‐user satisfaction was measured using the End‐User Computing Satisfaction questionnaire developed by Doll and Torkzadeh.^40^ The satisfaction questionnaire comprised 12 items addressing five components (content, accuracy, format, ease of use and timeliness) with 5‐point Likert scales (1 = strongly disagree, to 5 = strongly agree). We performed a translation from English to Korean using a committee approach.^41^ The Cronbach's *α* values of the five sections in this study were .824, .837, .683, .905 and .713. To assess usage, the variables of frequency of use, type of equipment used, the most useful function and experience of direct use during medical visits were measured only in the intervention group.

##### User experience of the APPSE

The key questions of the focus group interview were as follows: ‘How was your experience utilizing the application?’, ‘What do you think about patient participation using the application as it relates to patient safety?’, ‘What were your difficulties when using the APPSE?’, and ‘What are your suggestions for improving the application?’.

The open‐ended questions in the end‐user satisfaction survey asked which components of the APPSE were helpful, how difficult it was to use, what needed to be improved and what additional educational materials the respondent would like to receive.

### 2.6 Data collection

Participants were recruited between May 13 and June 20, 2018 from two websites, the Korea Alliance of Patients' Organizations (http://www.koreapatient.com/) and Resources for Enhancing Safety, Competency, and Utilization for Education (RESCUE; http://patientsafety.snu.ac.kr/), as well as through social media. The websites posted a description of the study and the link to the online survey. The pre‐ and post‐surveys were implemented using the Qualtrics online survey tool (https://www.qualtrics.com). A total of 131 participants completed the pre‐survey, and eligible participants were assigned to the intervention group receiving the APPSE programme or the control group. The ratio for randomization was 2:1 for the experimental and control groups. Random numbers for the intervention or control group were computer‐generated in a block size of 12 based on participants' gender and age, and then participants were randomly assigned using the Excel ‘RANDBETWEEN’ function. After random allocation, participants were informed of their assigned group by text message.

After 2 months of the intervention, the post‐surveys were performed with both the intervention and control groups using the online survey tool. The end‐user satisfaction survey was included in the post‐surveys of the intervention group. To recruit focus group participants among the post‐survey respondents in the intervention group, we sent text messages with a description of the focus group interview and the link to the online survey to respond for participation. The focus group interview was conducted in a seminar room at a university for about 2 h with five participants who agreed to participate. This study was approved by the Institute Review Board of Seoul National University (No. 1904/003‐003), and all study participants provided informed consent.

### 2.7 Statistical analysis

For the statistical analysis, the IBM SPSS V.24. programme was used. Homogeneity tests for demographics and general characteristics, and primary outcome variables at baseline between the intervention group and the control group were analysed using the independent *t*‐test and *χ* ^2^ test. For the comparison of primary outcome variables after the intervention, Mann–Whitney *U* tests were applied because the variables were not normally distributed. The false discovery rate (FDR < 0.2) procedure^42^ was carried out to adjust for multiple tests.

As secondary objectives of the study, satisfaction was analysed with descriptive statistics. To assess the user experience, the qualitative data from the focus group interview and open‐ended responses to the end‐user satisfaction survey were analysed by two researchers (N.‐J. L. and S. A.) using conventional content analysis^43^ as a qualitative descriptive approach.^44^ The focus group interview was audio‐recorded and transcribed. The field notes were used for analysis. Emerging themes were extracted using relevant quotes, and all the researchers discussed them in depth to ensure consensus on the themes.

## 3 RESULTS

**Figure 3:**
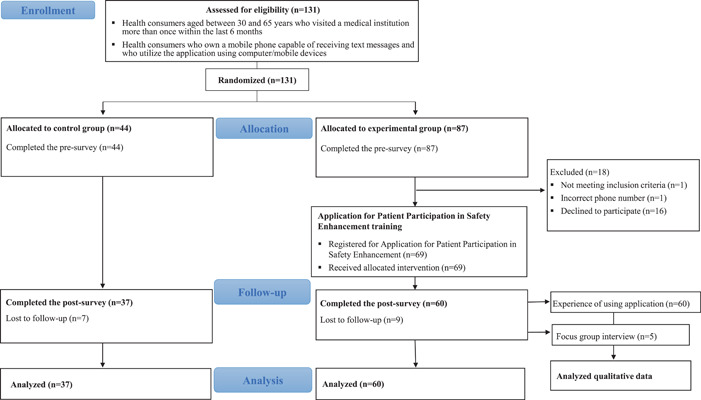
Flow diagram of participants

Participants' recruitment and retention are summarized in Figure 3. A total of 131 participants were randomly allocated to the intervention group (*n* = 87) or the control group (*n* = 44). Overall, 97 participants (74.0%) completed the post‐surveys.

### 3.1 Baseline data

There were no statistically significant differences in the demographic characteristics, variables related to health service use or primary outcome variables between the intervention and control groups. The baseline characteristics and pre‐measurement scores of the participants are shown in Table 2.

**Table 2:** Homogeneity tests between the experimental and control groups at baseline (*N* = 97)

Characteristics	Categories	Total (*N* = 97)	Experimental group (*n* = 60)	Control group (*n* = 37)	*t* or *χ* ^2^	*p*
		*n*(%) or *M* ± SD				
Age (years)	30–39	49 (50.5)	31 (51.7)	18 (48.6)	0.13	.938
	40–49	32 (33.0)	19 (31.7)	13 (35.1)		
	50–59	16 (16.5)	10 (16.7)	6 (16.2)		
Gender	Female	76 (78.4)	47 (78.3)	29 (78.4)	0.00	1.00
	Male	21 (21.6)	13 (21.7)	8 (21.6)		
Education	High school or below	13 (13.4)	8 (13.3)	5 (13.5)	0.94	.610
	Bachelor's degree	55 (56.7)	32 (53.3)	23 (62.2)		
	Master's degree or above	29 (29.9)	20 (33.3)	9 (24.3)		
Marital status	Single	26 (26.8)	17 (28.3)	9 (24.3)	0.12	.911
	Married	71 (73.2)	43 (71.7)	28 (75.7)		
Employment	Employed	77 (79.4)	50 (83.3)	27 (73.0)	1.50	.221
	Unemployed	20 (20.6)	10 (16.7)	10 (27.0)		
Monthly income (KRW)	<1,500,000	22 (22.7)	13 (21.7)	9 (24.3)	0.78	.676
	1,500,000–<4,500,000	60 (61.9)	39 (65.0)	21 (56.8)		
	4,500,000~	15 (15.5)	8 (13.3)	7 (18.9)		
Number of visits to medical institutions (last 6 months)	<5	59 (60.8)	40 (66.7)	19 (51.4)	2.29	.318
	5–<10	28 (28.9)	15 (25.0)	13 (35.1)		
	10~	10 (10.3)	5 (8.3)	5 (13.5)		
Reason for visiting medical institution	Acute disease	51 (52.6)	32 (53.3)	19 (51.4)	0.04	.849
	Chronic disease	46 (47.4)	28 (46.7)	18 (48.6)		
Number of chronic diseases	0	45 (46.4)	27 (45.0)	18 (48.6)	0.21	.898
	1	37 (38.1)	23 (38.3)	14 (37.8)		
	2~	15 (15.5)	10 (16.7)	5 (13.5)		
Type of medical institution frequently visited	Clinic	47 (48.5)	30 (50.0)	17 (45.9)	0.55	.758
	Hospital	15 (15.5)	8 (13.3)	7 (18.9)		
	General or advanced general hospital	35 (36.1)	22 (36.7)	13 (35.1)		
Experience of hospital admission (last 2 years)	Yes	25 (25.8)	16 (26.7)	9 (24.3)	0.07	.798
	No	72 (74.2)	44 (73.3)	28 (75.7)		
Experience of patient safety incidents	Yes	25 (25.8)	15 (25.0)	10 (27.0)	0.05	.825
	No	72 (74.2)	45 (75.0)	27 (73.0)		
Accompanying caregiver(s)	Alone	75 (77.3)	46 (76.7)	29 (78.4)	0.04	.845
	With caregiver(s)	22 (22.7)	14 (23.3)	8 (21.6)		
Experience of patient safety education	Yes	27 (27.8)	19 (31.7)	8 (21.6)	1.15	.284
	No	70 (72.2)	41 (68.3)	29 (78.4)		
Experience of patient participation education	Yes	12 (12.4)	7 (11.7)	5 (13.5)	0.07	.788
	No	85 (87.6)	53 (88.3)	32 (86.5)		
Patient safety knowledge			3.22 ± 0.76	3.15 ± 0.80	−0.43	.669
Self‐efficacy of participation			2.99 ± 0.61	2.83 ± 0.64	−1.20	.234
Willingness to participate			2.78 ± 0.55	2.69 ± 0.59	−0.79	.432
Experience of participation			2.24 ± 0.70	2.41 ± 0.74	1.14	.256

### 3.2 Primary outcomes

Among the results of the comparisons of the post‐survey data between the intervention and the control groups, the intervention group had significantly higher scores overall than the control group with regard to patient safety knowledge (*U* = 646.00, *p* < .001), self‐efficacy of participation (*U* = 679.00, *p* = .001), willingness to participate (*U* = 767.50, *p* = .010) and experience of participation (*U* = 833.00, *p* = .038; Table 3). Compared with the control group, the patient safety activities with significantly higher scores in both the self‐efficacy and willingness sections were 7 activities among 13 activities (FDR < 0.2) (Table S1).

**Table 3:** Patient safety knowledge, self‐efficacy of participation, extent of willingness to participate and experience of participation in patient safety activities after participation in the programme (*N* = 97)

Outcomes	Groups (*n*)	Mdn (IQR)	*M* ± SD	Mean difference	*U*	*p* value	*r*	*d*
Patient safety knowledge	Experimental (*n* = 60)	3.88 (0.79)	3.77 ± 0.67	0.48 ± 0.14	640.00	<.001	0.36	0.74
	Control (*n* = 37)	3.25 (0.79)	3.28 ± 0.66					
Self‐efficacy of participation	Experimental (*n* = 60)	3.23 (0.44)	3.13 ± 0.50	0.32 ± 0.11	679.00	.001	0.33	0.61
	Control (*n* = 37)	2.92 (0.77)	2.81 ± 0.56					
Willingness to participate	Experimental (*n* = 60)	3.00 (0.60)	2.95 ± 0.53	0.27 ± 0.11	767.50	.010	0.26	0.52
	Control (*n* = 37)	2.69 (0.77)	2.68 ± 0.50					
Experience of participation	Experimental (*n* = 60)	1.77 (0.92)	1.88 ± 0.83	0.33 ± 0.16	833.00	.038	0.21	0.43
	Control (*n* = 37)	1.31 (0.88)	1.55 ± 0.64					

*Note*: *r *= |*Z*/√*N*|.

Abbreviations: *d*, Cohen's *d*; IQR, interquartile range; Mdn, median; *r*, effect size *r*; *U*, Mann–Whitney *U*.

### 3.3 Secondary outcomes

#### 3.3.1 End‐user satisfaction

The total mean end‐user satisfaction score was 3.56 ± 0.60 (Table 4). The three high‐score categories were accuracy (3.71 ± 0.77), content (3.64 ± 0.54) and format (3.60 ± 0.70). The mean scores of the ease of use and timeliness categories were 3.26 ± 0.97 and 3.53 ± 0.71, respectively. Of the participants, 95% used the system at least once a week. All participants used the APPSE by mobile or web app, and most participants (90.0%) used mainly a mobile phone or a tablet PC to utilize the app. The responses to the most useful function in the system were ‘SAFE educational materials’ (36.7%), ‘Listing my medications’ (23.3%) and ‘Engaging in patient safety activities’ (18.3%).

**Table 4:** Evaluation of end‐user satisfaction of the APPSE (*N* = 60)

Variables	Categories	*M* ± SD
Content		3.64 ± 0.54
	Does the system provide the precise information you need?	3.60 ± 0.62
	Does the information content meet your needs?	3.80 ± 0.63
	Does the system provide reports that seem to be just about exactly what you need?	3.63 ± 0.71
	Does the system provide sufficient information?	3.53 ± 0.70
Accuracy		3.71 ± 0.77
	Is the system accurate?	3.75 ± 0.88
	Are you satisfied with the accuracy of the system?	3.67 ± 0.77
Format		3.60 ± 0.70
	Do you think the output is presented in a useful format?	3.45 ± 0.95
	Is the information clear?	3.75 ± 0.63
Ease of use		3.26 ± 0.97
	Is the system user friendly?	3.25 ± 1.04
	Is the system easy to use?	3.27 ± 0.99
Timeliness		3.53 ± 0.71
	Do you get the information you need in time?	3.45 ± 0.85
	Does the system provide up‐to‐date information?	3.62 ± 0.76
Total		3.56 ± 0.60

Abbreviation: APPSE, Application for Patient Participation in Safety Enhancement.

#### 3.3.2 User experience of the APPSE

All participants of the intervention group answered the open‐ended responses to the end‐user satisfaction survey. Five health consumers participated in the focus group interview. Four of the interviewees were female and one was male, and the average age was 45.6 years. The qualitative data were categorized as experiences with the intervention and suggestions for improving the APPSE (Table 5).

**Table 5:** Patients' experiences with the intervention of using the APPSE and suggestions for improvement

Theme	Quotes
***Patients' experiences with the intervention***	
Providing systematic material	It was good for me that specific and necessary information for health consumers was organized in the educational content. (End‐user satisfaction survey, Participant 13)
	Some of the educational content was what I'd already known and some of the contents were new things. The best thing about participating in this intervention was that the APPSE was a helpful application, in that it consisted of systematic content on patient participation. (FGI, Participant 4)
Being informed	The APPSE provides useful information about patient safety and patient participation. (End‐user satisfaction survey, Participant 5)
	This application helps me to understand patient safety better and how to participate in it. It is informative knowledge that is not easily acquired elsewhere. (End‐user satisfaction survey, Participant 12)
Understanding various aspects of participation	
Records of my medications	It was helpful to me to see my medication list all at once. (End‐user satisfaction survey, Participant 37)
Patient's rights	I learned about some things that I didn't think about being part of patient safety, such as a patient's rights. (End‐user satisfaction survey, Participant 31)
Question list	I have prepared what to do and questions to ask before seeing my doctor today. I made a list of questions with the first question to ask and the second question to ask, and it was helpful. (FGI, Participant 2)
Hand washing	I wasn't aware of healthcare providers' handwashing before, but I learned that it is an important part of patient safety. (End‐user satisfaction survey, Participant 41)
Bringing a patient advocate	I think selecting a patient's advocate is good for patients, but I have continued to go to the hospital alone because in my situation my caregiver is not able to accompany me. But I believe it is necessary for an advocate to accompany a patient. (FGI, participant 5)
Linking patient participation to patient safety	In the meantime, even though I was doing it in the hospital, I never realized that what I was doing was patient participation. After using the APPSE, I realized that my actions were patient safety activities, and it is necessary for my safety in my care process. I felt that patient safety is broader than I had thought. (FGI, Participant 2)
Awareness of the need to spread patient participation training	I hope education for improving patient participation like this program becomes more widespread. (FGI, Participant 4)
	I think that if [a positive] perception of patient participation and education for health consumers is established, patient safety accidents and medical costs can be reduced. (FGI, Participant 5)
Transition to active patient	I tried to pay attention to healthcare providers' attitudes and behaviours before and during my treatment that I could have overlooked before. Also, I insisted on a more precise answer to my questions. (End‐user satisfaction survey, Participant 40)
	I keep thinking about which questions I can ask about my treatment using my question list. When I kept asking questions, I felt that communication with the doctor was better than before. (FGI, Participant 2)
	As a patient, I think it would be important for me to change my passive behaviours to active behaviours. (End‐user satisfaction survey, Participant 6)
Difficulty with attempts at participation	It seemed that the healthcare provider didn't wash their hands, but it was difficult to ask if they had washed their hands. (FGI, Participant 2)
	It's not so easy to ask the doctor questions. So, I wrote down a list of questions before I went to see the doctor. There were five questions I would have liked to ask, but when I was asking the third question, the doctor didn't pay attention to me, and he seemed too busy. So, I wasn't able to ask any more. (FGI, Participant 5)
***Patients' suggestions on how the APPSE could be improved***	
More user‐friendly interface	Sometimes there was the problem that the system's user interface did not work properly. It should be improved to be more user‐friendly and easier to use. (End‐user satisfaction survey, Participant 3)
	I usually don't use the internet, so the app was unfamiliar to me at first. When I first used it, I didn't know how to log in and couldn't see all the contents of the application. (FGI, Participant 5)
Additional communication routes between users and the system administrator	I think, if there were a bulletin board where I can ask the system administrators questions while learning the educational content, I would be able to share my opinions and learn more. (End‐user satisfaction survey, Participant 13)
Adding graphics to assist comprehension	It would be nice to insert pictures or examples to help users understand the content. (End‐user satisfaction survey, Participant 29)
	I felt complicated to read because there was too much information on one mobile screen. It is necessary to add pictures or graphics and simplify the list. (End‐user satisfaction survey, Participant 59)
A brief summary of educational information	There is a lot of information to be conveyed, and it has to be explained in detail so that health consumers can understand it. So, it seems that it must have been difficult to organize the educational content. I think it is very important to think about a simpler layout that is more accessible and provide a summary of information and information that can be found more easily. (End‐user satisfaction survey, Participant 44)
Using simpler terms	Change to easy terms that can be easily understood. (End‐user satisfaction survey, Participant 26)
Connecting the APPSE with hospital systems or the national reporting system	Provide a notification service linked to the hospital visit schedule. (End‐user satisfaction survey, Participant 14)
	Connect to the national medical error reporting system. (End‐user satisfaction survey, Participant 8)

Abbreviations: APPSE, Application for Patient Participation in Safety Enhancement; FGI, focus group interview.

The participants reported that the information provided by the system was specific, and the system provided the necessary information systematically to the patient. They learned various aspects of patient participation that they did not know before taking part in this study, which gave them a new perspective on patient participation in patient safety. After participating in the intervention, the participants mentioned a change in their perceptions of patient participation, and they reported changes in behaviours towards being more active patients during hospital visits. The participants also mentioned their experience of patient safety activities such as making a medication list and question list; after participating in the intervention, participants used the tools provided by the system to check their medications and questions to ask and felt that these activities could improve patient safety. In addition, they realized that patients could play an important role in improving patient safety by participating in safety‐related activities in their care process. On the other hand, there were obstacles to the patients' participation in their care process in the hospital. Participants felt that their healthcare providers seemed so busy that they could not talk with the patient. The time constraints between patients and healthcare providers and the patient's passive attitude were major factors that made participation difficult.

The participants' suggestions for improving the APPSE were classified into six themes. The suggestions included a more user‐friendly interface, additional communication routes between users and the system administrator, adding graphics and simpler terms to ease understanding, providing a brief summary of educational information, and connection with hospital systems or a national error reporting system.

## 4 DISCUSSION

Patient participation is a crucial element in improving patient safety and care quality. To enable patient participation, patients should be provided with relevant information and education. Patients who are more knowledgeable about patient safety are more likely to participate in patient safety activities and their care.^20^, ^21^, ^27^ In addition, programmes and various other patient safety education resources such as leaflets, patient safety videos or patient‐safety campaigns targeting patients positively affect their attitudes towards and behaviours of patient participation.^10^, ^27^, ^45^ In this study, we developed a mobile application to encourage health consumers' participation in patient safety activities by providing information on patient participation and actionable tools for participation in their care process, and evaluated the effects of the mobile application. Participants in this study demonstrated significant improvement in patient safety knowledge, self‐efficacy of participation, willingness to participate and experience of participation in patient safety activities. The qualitative data from the semi‐structured interviews and open‐ended responses to the end‐user satisfaction survey support the findings from the quantitative section.

As a patient safety principle, patient participation applies throughout the patient's healthcare process,^46^ and information sharing with patients is a way to promote participation. Previous research reported that patients were willing to participate in an intervention designed to support them in collaboration with healthcare professionals to improve patient safety.^13^ Thus, when developing an educational programme to improve patient participation, it is necessary to consider patients' needs, preferences and motivation to participate.^13^, ^15^, ^47^ Therefore, in this study, the APPSE was developed through a health consumer needs assessment and expert review of educational content. After analysing health consumers' needs, we systematically added various topics that patients could experience in the hospital based on a competency‐based framework through expert review.

Mobile‐based education has several advantages compared with traditional educational methods such as the didactic method for conveying information on patient safety or patient participation. Patients can easily access educational information at any place and at any time.^48^ South Korea, in particular, is one of the most developed countries with regard to information technology.^20^ According to a national survey in South Korea, 44.3% of online education is accessed on desktop devices and 47.2% on mobile devices.^23^ For this reason, mobile‐based education could be an effective method of patient education.^20^

Patients' extent of knowledge of healthcare has an important influence on patient participation.^15^ In the current study, health consumers' patient safety knowledge score was significantly different between the intervention and control groups. This result was consistent with previous findings.^20^ In the study of Cho and Lee,^20^ which explored the effect of using a smartphone application for patient education, they found that surgical patients' knowledge about safety issues was significantly improved. Most patients are not able to obtain information quickly, and patients often remember limited medical information.^48^ However, using an effective mobile application for education, patients can learn repeatedly as often as they want.

This study demonstrated that the APPSE brought about significant improvement in a health consumer's self‐efficacy, willingness to participate and experience of participation. Most previous studies of patient safety education for patient participation partially assessed patient safety competence changes such as safety knowledge,^20^ perception and attitudes,^10^, ^16^ or safety‐related behaviours and experiences of safety‐related incidents^27^ after training, but our study attempted to assess all components of patient safety competence, including knowledge, perception and experience at the patient level.

Promoting awareness of patient safety issues and competency in participation is important because it increases patients' chances of participating in patient safety activities. Among patient safety activities, previous studies have consistently reported that patients are unwilling to ask healthcare workers questions that challenge their authority, such as whether they had washed their hands.^27^, ^49^ Although patients know the importance of hand hygiene to prevent infection, the patients were unable to directly ask healthcare workers to do so.^20^ According to Lee et al.,^49^ almost 90% of the Korean general public who participated in their study perceived patient safety as an important issue, but their self‐efficacy of patient safety and participation was relatively low. Unlike previous studies,^20^, ^27^, ^29^ self‐efficacy of participation and the extent of willingness improved significantly after the intervention including challenging patient safety activities in this study. However, the average score for experience of participation after the intervention was lower than those for self‐efficacy of participation and the extent of willingness. There was no significant difference in the individual items in the experience of patient participation between the experimental group and the control group after controlling for the FDR.

The qualitative data of this study identified potential obstacles that lead to difficulties in participating patient safety. Healthcare providers' interactions or relationships with patients can affect participation in safety activities. Hierarchical relationships between healthcare providers and patients, failure to share treatment plans or decision‐making processes with patients, and healthcare providers' disinterest or negative reactions can be barriers to patient participation.^3^, ^15^, ^50^, ^51^ In our study, we attempted to promote participation in patient safety activities by asking questions to the healthcare providers or speaking up about concerns through the application. However, if healthcare providers react negatively to these challenging behaviours and their reactions are reflected in the treatment process, that may be a factor that reduces the use of this application and ultimately diminishes patient participation. Therefore, to promote patient participation in safety activities, it is necessary for healthcare providers to communicate and interact more with patients, as well as to build a supportive safety culture based on a positive belief in patient participation. Future research is also needed to encourage patients to use the APPSE while visiting the hospital and to measure its effects over a long‐term intervention period to change patient participation behaviours.

### 4.1 Strengths and limitations

The strength of this study is that the APPSE provided actionable tools to enable the user to participate in their care process when visiting the hospital, beyond simply providing information. For example, the APPSE provided patient safety activities that patients could participate in while they were at the hospital and check off after performing the activities directly in the app. To improve patient participation, it is important to reduce the gaps among health consumers' knowledge of participation and intention to participate, and, on the other hand, actual participation behaviours. Educational materials and patient participation tools could narrow these gaps and motivate health consumers' health‐related behaviour. A previous intervention study on patient safety included a quiz function on patient safety in a mobile application to test participants' knowledge level.^20^ In this study, the APPSE increased participants' experience of patient participation and enhanced their knowledge as a tool constructed to help health consumers understand relevant information and participate in patient safety activities. It is especially notable that participants in this study reported that they have changed from being a passive patient to an active patient through the experience of using the ‘Listing my medications’, ‘Asking questions’ and ‘Engaging in patient safety activities’ functions. In future studies, to help patients understand and to increase the utilization of the mobile application, the application needs to be improved by adding more graphics, creating a communication route between users and the system administrator and using simpler terminology.

This study has several limitations. First, we could not include all participants who had received the intervention in the focus group interviews because the participation rate was low due to online recruitment or short intervention periods. Second, the sample was recruited through two websites and social media, so those who were more interested in this subject or those who regularly used computers or mobile apps would be more likely to participate in this study. According to the national data reported by the Korea Institute for Health and Social Affairs,^52^ individuals aged 60 or over were the age group that accounted for the highest proportion of annual hospital visits (at 32.7%), and those with a high school diploma or less accounted for the highest proportion (at 48.8%). Considering this statistic, the participants of our study might be a relatively young and highly educated population compared to the segments of the population that account for the most hospital visits. We randomized the group assignment to reduce the selection bias of participants who were interested in patient safety. However, these characteristics of the participants may have affected the outcomes, and the results may not be generalizable to all patient groups. Third, we could not adjust for the participating health consumers' diverse illness‐related characteristics and individual characteristics that potentially affected the extent of changes in their patient safety willingness and experience. Fourth, even though the educational contents were developed based on the findings of the health consumer needs assessment and a systematic literature review, they were validated only by healthcare professionals. In the focus group interview conducted after the intervention, the participants expressed the opinion that it would be better to use simple terms and use more graphics to increase patients' understanding. Therefore, the future development process should consider involving patients in the content verification process, which may enable the development of content that patients can understand more easily by considering their health literacy. Future research should evaluate the intervention with a more diverse population and track long‐term outcomes.

## 5 CONCLUSION

Mobile applications may enable more active participation of health consumers by providing information and tools for participation in patient safety activities. This study demonstrates that educating health consumers through a mobile application with relevant information improves their level of patient safety knowledge, self‐efficacy of participation, the extent of willingness to participate and experience of participation in patient safety activities. Healthcare providers can play a crucial role in encouraging patients to participate in safety activities by providing patient education using a mobile application, which is an important step toward building patient‐centred care in the healthcare system. Further intervention studies with the APPSE or efficient patient safety education applications are needed to assess the ongoing effectiveness of mobile applications for patient participation, for improving patient safety outcomes.

## Supporting information

Supporting information.

## Data Availability

The data are not publicly available due to privacy or ethical restrictions.
