# Supplementary material for: The effects of a mobile application for patient participation to improve patient safety
Source: Health Expect. 2022 May 11;25(4):1601–18. doi: 10.1111/hex.13503 (PMC9327837; doi:10.1111/hex.13503)
Supplement: Supplementary file 1 — Supporting information. [file HEX-25--s001.docx]

Supplementary table 1. Self-efficacy of participation, extent of willingness to participate, and experience of participation in patient safety activities after participation in the program (*N*=97)

| Patient participation practices | Self-efficacy of participation | | | Willingness to participate | | | Experience of participation | | |
| --- | --- | --- | --- | --- | --- | --- | --- | --- | --- |
|  | Experimental  group  (*n*=60) | Control  group  (*n*=37) | Mann–Whitney *U*-test | Experimental  group  (*n*=60) | Control  group  (*n*=37) | Mann–Whitney *U*-test | Experimental  group  (*n*=60) | Control  group  (*n*=37) | Mann–Whitney *U*-test |
|  | M±SD | | *FDR-adjusted p value* | M±SD | | *FDR-adjusted p value* | M±SD | | *FDR-adjusted p value* |
| Seeking a second opinion regarding an important healthcare decision | 3.10±0.60 | 2.59±0.80 | .007* | 2.70±0.74 | 2.49±0.80 | .278 | 1.93±1.01 | 1.41±0.76 | .568 |
| Asking healthcare workers to explain more fully something they just said that I do not understand | 3.13±0.75 | 2.84±0.83 | .107* | 3.05±0.77 | 2.68±0.75 | .084* | 2.42±1.17 | 1.70±0.88 | .568 |
| Bringing a friend or family member to a doctor’s appointment so that they can help ask questions and understand what the doctor was telling me | 2.93±0.76 | 2.76±0.72 | .028 | 2.33±0.84 | 2.19±0.81 | .456 | 1.72±1.03 | 1.49±0.73 | .714 |
| Asking healthcare workers if they washed their hands | 2.58±0.89 | 2.16±0.87 | .081* | 2.12±0.92 | 1.68±0.82 | .084* | 1.50±0.93 | 1.27±0.65 | .714 |
| Telling healthcare workers about any drug allergies when they did not ask for this information | 3.50±0.68 | 3.08±0.64 | .007* | 3.47±0.79 | 3.14±0.71 | .084* | 1.90±1.20 | 1.59±1.01 | .650 |
| Asking healthcare workers to confirm my identity before performing a procedure | 3.12±0.76 | 2.73±0.77 | .049* | 2.88±0.96 | 2.68±0.91 | .286 | 1.87±1.23 | 1.38±0.83 | .568 |
| Asking healthcare workers about the details of a procedure and the reason for a procedure before it is performed | 3.37±0.64 | 3.14±0.67 | .107* | 3.37±0.69 | 3.16±0.73 | .245 | 1.97±1.28 | 1.76±1.19 | .650 |
| Asking healthcare workers to explain care, such as an X-ray or drawing blood, that I was not told about by my doctor or nurse | 3.25±0.75 | 2.92±0.72 | .049* | 3.25±0.84 | 2.95±0.74 | .084* | 1.80±1.18 | 1.49±0.84 | .568 |
| Calling a healthcare worker when I undergo medical tests ordered and no one calls me with the results | 3.40±0.59 | 3.08±0.83 | .107* | 3.42±0.72 | 3.11±0.74 | .084* | 1.70±1.11 | 1.57±1.07 | .568 |
| Taking a written list of all the medications I’m currently taking when going to the doctor | 3.17±0.81 | 2.84±0.90 | .097* | 3.00±0.92 | 2.62±0.89 | .084* | 1.92±1.24 | 1.84±1.21 | .568 |
| Questioning medications or pills if I did not recognize them and never took this medication in the past | 3.22±0.78 | 2.97±0.69 | .097* | 3.17±0.91 | 2.86±0.71 | .084* | 2.02±1.24 | 1.59±0.93 | .650 |
| Checking that I received the right drug and strength before leaving the pharmacy | 3.13±0.77 | 2.86±0.92 | .167* | 3.07±0.86 | 2.78±1.00 | .278 | 2.40±1.32 | 1.86±1.13 | .781 |
| Reporting the errors I noticed had occurred in the hospital to a national reporting system | 2.82±0.77 | 2.57±0.83 | .167* | 2.52±0.87 | 2.54±0.84 | .948 | 1.32±0.81 | 1.19±0.62 | .559 |

*Note:* * significant at False Discovery Rate (FDR)<0.2.
